# Fusion Proteins CLD and CLDmut Demonstrate Potent and Broad Neutralizing Activity against HIV-1

**DOI:** 10.3390/v14071365

**Published:** 2022-06-23

**Authors:** Ming Fu, Yingying Xiao, Tao Du, Huimin Hu, Fengfeng Ni, Kai Hu, Qinxue Hu

**Affiliations:** 1 State Key Laboratory of Virology, Wuhan Institute of Virology, Center for Biosafety Mega-Science, Chinese Academy of Sciences, Wuhan 430071, China; fuming199142@foxmail.com (M.F.); xiaoyingying0622@126.com (Y.X.); dut@wh.iov.cn (T.D.); hhm_gzyx@163.com (H.H.); nifengfeng1201@163.com (F.N.); 2008hukai@live.cn (K.H.); 2 Savaid Medical School, University of Chinese Academy of Sciences, Beijing 100049, China; 3 Institute for Infection and Immunity, St George’s, University of London, London SW17 0RE, UK

**Keywords:** HIV-1, CD4, DC-SIGN, CLD, CLDmut, bNAbs

## Abstract

HIV-1 envelope glycoprotein (Env) interacts with cellular receptors and mediates virus entry into target cells. Blocking Env-receptor interactions represents an effective interventional strategy for developing HIV-1 entry inhibitors. We previously designed a panel of CD4-linker-DC-SIGN (CLD) constructs by fusing the extracellular CD4 and DC-SIGN domains with various linkers. Such CLDs produced by the prokaryotic system efficiently inhibited HIV-1 infection and dissemination in vitro and ex vivo. In this study, following the construction and identification of the most promising candidate with a linker of 8 Gly_4_Ser repeats (named CLD), we further designed an improved form (named CLDmut) by back mutating Cys to Ser at amino acid 60 of CD4. Both CLD and CLDmut were produced in mammalian (293F) cells for better protein translation and modification. The anti-HIV-1 activity of CLD and CLDmut was assessed against the infection of a range of HIV-1 isolates, including transmitted and founder (T/F) viruses. While both CLD and CLDmut efficiently neutralized the tested HIV-1 isolates, CLDmut demonstrated much higher neutralizing activity than CLD, with an IC_50_ up to one log lower. The neutralizing activity of CLDmut was close to or more potent than those of the highly effective HIV-1 broadly neutralizing antibodies (bNAbs) reported to date. Findings in this study indicate that mammalian cell-expressed CLDmut may have the potential to be used as prophylaxis or/and therapeutics against HIV-1 infection.

## 1. Introduction

Antiretroviral therapy (ART) has transformed HIV-1 infection from a fatal disease to a chronic disease that can be controlled by drugs [1,2]. However, such therapies do not clear the virus, and life-long medication is required. At present, most anti-HIV-1 drugs are chemicals which cause serious and complex side effects and drive the emergence of drug-resistant HIV-1 variants [3,4]. While biological medicines against the virus may have the advantage of low toxicity and the potential to be persistently expressed in patients [5,6,7], currently, only the fusion inhibitor Enfuvirtide and its analogs have been approved for clinical use [8,9]. Although bNAbs have shown promising results in animal challenge studies and early clinical trial studies [10,11,12], they are very expensive to produce and unlikely to be used widely even if the trials are successful. Therefore, novel biological intervention strategies are needed.

The binding of the envelope glycoprotein (Env) gp120 to CD4 is virtually universal among HIV-1 isolates. CD4 is the primary receptor for HIV-1 entry and can mediate the transmission of HIV-1 from cell to cell. HIV-1 entry is initiated by binding the CD4 to the viral envelope glycoprotein (Env) gp120 [13,14]. Soluble CD4 (sCD4) can act as a receptor decoy to prevent the engagement of HIV-1 Env with cell-surface CD4, and it has been shown that sCD4 containing the first two extracellular domains (D1D2) is a potent inhibitor of HIV-1 entry [15]. DC-SIGN is highly expressed on dendritic cells (DCs) residing in mucosal tissues, which can capture HIV-1 and facilitate HIV-1 trans-infection of permissive cells [16]. The extracellular domain consists of the neck domain (ND) and carbohydrate recognition domain (CRD). The ND of DC-SIGN has been reported to lead to tetramerization through hydrophobicity, resulting in ND changing from β-rotation to α-superhelix, which can enhance protein affinity to corresponding ligands [17]. CRD contains ligand-binding sites that bind two calcium ions and one mannose. Several studies have shown that antagonists against DC-SIGN inhibit DC-SIGN-mediated HIV-1 transmission. In this regard, we previously designed a series of CD4 (D1D2)-linker-DC-SIGN (ND and CRD) fusion proteins (CLDs) that target both CD4 and DC-SIGN binding sites on the HIV-1 Env, with a (Gly_4_Ser)n linker ensuring that both components of the fusion protein can access their binding sites [18]. Compared to soluble CD4 (sCD4), CLDs produced from prokaryotic cells exhibited significantly enhanced neutralizing activity against various HIV-1 isolates and potently prevented both localized and disseminated infections of HIV-1 in mucosal tissues [18]. CLDs have a much slower off-rate than sCD4 and facilitate the formation of a more stable CD4-Env complex, rendering them with higher gp120-binding avidity and a better anti-HIV potency [18,19]. Because the soluble forms of CD4 and DC-SIGN occur normally in vivo and the Gly_4_Ser repeat linker is poorly immunogenic [20,21,22], further improvement of CLDs may make them potentially useful as prophylactics such as topical microbicides or/and as therapeutic agents.

However, the above CLD fusion proteins expressed by *E. coli* mainly existed in the form of inclusion bodies [23]. Although they showed high anti-HIV-1 activity after refolding, a certain degree of incorrect formation of disulfide bonds or/and aggregation occurred. Therefore, in this study, we used mammalian cells to express the fusion proteins. Given that the linker length could affect the anti-HIV-1 activity of CLDs, probably by interfering with CLD-gp120 interactions [18], we designed and screened CLD fusion proteins with a linker of 7, 8, or 9 Gly_4_Ser repeats and found that the CLD with 8 Gly_4_Ser repeats (named CLD) was the most potent. The Ser-to-Cys mutation at amino acid 60 in fusion protein CLDs expressed by *E. coli* was originally introduced to improve the interaction with gp120 [24]. Considering that mammalian cells express proteins that undergo complete post-translational modification, such mutation may not be essential, and, therefore, we back mutated Cys to Ser and designated the construct CLDmut. Both CLD and CLDmut containing 8 Gly4Ser repeats were subsequently produced in the mammalian 293F cells, and their anti-viral activities were assessed against a wide range of HIV-1 isolates, including T/F viruses. We also compared the neutralizing activities of CLD and CLDmut with those of a panel of highly effective HIV-1 bNAbs. Findings in this study highlight the potential of the mammalian cell-expressed CLDmut to be developed as an effective and broadly neutralizing biological agent against HIV-1 infection.

## 2. Materials and Methods

### 2.1. Cells

The 293T cell line was purchased from the American Type Culture Collection (ATCC). The TZM-bl cell line was obtained from the NIH AIDS Research and Reference Reagent Program. Both cell lines were grown in Dulbecco modified Eagle medium (DMEM) with 10% fetal bovine serum (FBS, Thermo Scientific, Sydney, Australia) at 37 °C with 5% CO_2_. The FreeStyleTM 293F cell line was purchased from ThermoFisher Scientific and grown in FreeStyleTM 293 Expression Medium at 37 °C with 5% CO_2_ in a shaking incubator. All media were supplemented with 100 U/mL penicillin/streptomycin (Genom, Hangzhou, China).

Human peripheral blood mononuclear cells (PBMCs) were isolated from healthy adult donors using Human Peripheral Blood Lymphocyte Isolates (TBD Science, Tianjin, China), followed by stimulation with PHA (1 µg/mL) and maintained in a medium supplemented with recombinant human IL-2 (20 U/mL) for 7 days. PBMCs were grown in RPMI 1640 medium supplemented with 100 U/mL penicillin/streptomycin (Genom, Hangzhou, China) at 37 °C with 5% CO_2_.

### 2.2. HIV-1 Production

All T/F HIV-1 plasmids were obtained from NIH AIDS Research and Reference Reagent Program. Subtype B strains and subtype C strains CH164, CH185, CH198, and CH811 were produced in 293T cells transfected with corresponding full-length infectious HIV-1 clones in plasmids. Other subtype C strains were Env-pseudotyped viruses, produced by co-transfecting 293T cells with p-gp160 and the HIV-1 backbone plasmid pSG3ΔEnv in a ratio of 1:3 using Lipofectamine 2000 (Life Technology, Carlsbad, CA, USA). A total of 48 h post-transfection, the virus-containing medium was harvested and filtrated through a 0.45 µm filter, aliquoted, and stored at −80 °C. The virus stocks were titrated to measure 50% tissue culture infection doses (TCID_50_) in TZM-bl cells. HIV-1 stocks of laboratory-adapted isolates BaL and NL4-3 were prepared as described previously [25,26,27].

### 2.3. Design and Genetic Engineering of Protein Expression Constructs

CLD constructs in pET28a(+) were described previously [18], which contain the D1D2 domain of human CD4 (residues 1–178) and the neck domain (ND) and carbohydrate-recognition domain (CRD) of human DC-SIGN (residues 88–404). In this study, a Cys-to-Ser back mutation was introduced to the amino acid 60 of CD4 to make one construct CLDmut. In both CLD and CLDmut, a linker of 8 Gly_4_Ser repeats was used to replace the linker of 7 Gly_4_Ser repeats. Human codon-optimized CLD and CLDmut genes were inserted into pcDNA3.1(+) (Invitrogen, Carlsbad, CA, USA), respectively. The signal peptide sequence was MDRAKLLLLLLLLLLPQAQA, and the his-tag was located between the signal peptide sequence and CD4-D1D2 sequence. All recombinant DNA clones were confirmed by sequencing.

### 2.4. Expression and Purification of CLD and CLDmut Proteins

We used 293F cells to produce CLD and CLDmut proteins, as previously described with modifications [28]. In brief, 1 million cells were transfected with 1–1.2 µg protein-expressing plasmids using 4 µg polyethyleneimine (PEI). Four days post-transfection, cell culture supernatants were harvested for purification. The his-tagged CLD and CLDmut proteins were purified by Ni-NTA (GE Healthcare, Oslo, Norway) followed by gel-filtration chromatography on SuperdexTM 75 Increase 10/300 GL. Protein concentration was determined by BCA assay according to the manufacturer’s instructions (Takara Bio, Beijing, China).

### 2.5. SDS-PAGE, Native PAGE and Western Blot

These experiments were performed as described previously [18]. For SDS-PAGE, cell culture supernatants or purified proteins were boiled for 10 min with loading buffer (50 mM Tris-HCl, pH 6.8, 2% SDS, 25% glycerol, 1% DTT), and the samples were separated by 10–12% SDS-PAGE. For BN-PAGE, purified protein samples were prepared in 2 × Protein Native PAGE Loading Buffer (TaKaRa, Dalian, China) and loaded onto a 4 to 12% Bis-Tris NuPAGE gel (Invitrogen, Carlsbad, CA, USA). Western blot analysis was performed as described previously [18]. The proteins were transferred to a polyvinylidene difluoride (PVDF) membrane (0.45 µm; Millipore, Cork, Ireland). PVDF membrane was blocked with 5% nonfat milk and subsequently incubated with an anti-DC-SIGN MAb (Santa Cruz, clone 120507, Dallas, TX, USA) for 1 h at room temperature. After 3 washes with TBS-Tween (200 mM NaCl, 50 mM Tris-HCl, 0.1% Tween-20), the membrane was incubated with a horseradish peroxidase (HRP)-conjugated goat anti-mouse antibody (ThermoFisher Scientific, Waltham, MA, USA) for 1 h at room temperature. Protein bands were visualized following incubation with enhanced chemiluminescence (ECL) (Millipore, Billerica, MA, USA).

### 2.6. HIV-1 Neutralization Assay

For the neutralization assay performed on the TZM-bl cell line, 5 × 10^3^ cells were seeded in 96-well plates. Serially diluted CLD, CLDmut, or the indicated bNAb (obtained from the NIH AIDS reagent program) were incubated with 200 TCID_50_ HIV-1 at 37 °C for 1 h, and the mixtures were added to the cells. Following 48 h cultivation, the cells were lysed using cell lysis buffer (Beyotime, P0013, Shanghai, China), and the luciferase activity of cell lysates was measured using a Modulus Microplate Luminometer (Turner BioSystems, Sunnyvale, CA, USA). Background luciferase activity from uninfected cells was subtracted. The 50% inhibitory concentration (IC_50_) values were calculated using GraphPad Prism software (version 7.0, San Diego, CA, USA).

For the neutralization assay performed in PBMCs, 2 × 10^4^ cells were seeded in 96-well plates. Serially diluted CLD or CLDmut were incubated with 200 TCID_50_ HIV-1 at 37 °C for 1 h, and the mixtures were added to the cells. Following 3 h incubation, the supernatants of PBMCs were removed, and the cells were extensively washed with PBS. Three days post-infection, the supernatants were harvested and lysed using 0.1% Triton X-100 (Sigma-Aldrich, Shanghai, China) at 37 °C for 1 h. The p24 concentration was measured using an enzyme-linked immunosorbent assay (ELISA) according to the manufacturer’s instructions (KeYuanAnBo, Wuhan, China). The optical density values were measured at 450 nm (test wavelength) and 620 nm (reference wavelength) using an ELISA plate reader (Tecan, Männedorf, Switzerland). The 50% inhibitory concentration (IC_50_) values were calculated using GraphPad Prism software (version 7.0, San Diego, CA, USA).

### 2.7. Bio-Layer Interferometry (BLI)

CN54 gp140 was described previously [27]. CLDmut protein was conjugated with biotin at a molecular molar ratio of 1:3. A total of 5 µg/mL biotinylated CLDmut protein was coupled to Octet^®^ Streptavidin (SA) biosensors (No. 18-5019) and immersed in different concentrations of CN54 gp140 (50, 25, 12.5, or 6.25 nM), obtained from the NIH AIDS reagent program, for 600 s, and, then, the baseline interference was read for 600 s in kinetics buffer (KB: 1 × PBS, 0.05% BSA, 0.02% Tween 20, pH 7.4). The anti-HIV-1 broadly neutralizing antibody 3BNC117 was directly coupled to Octet^®^ Anti-Human Fc Capture (AHC) biosensors (No. 18-5060) [29] and immersed in different concentrations of CN54 gp140 (50, 25, 12.5, or 6.25 nM) for 600 s, and, then, the baseline interference was read for 600 s in kinetics buffer (KB: 1 × PBS, 0.05% BSA, 0.02% Tween 20, pH 7.4). All kinetic interactions were measured with new sensors at 30 °C and 1000 rpm in 96-well plates under an Octet Red96 system (Pall ForteBio, Fremont, CA, USA). Curve fitting was done with a 1:1 binding model using the ForteBio software (Data Acquisition 7.1, Sartorius, Göttingen, Germany). Mean K_on_ and K_off_ and apparent KD values were calculated from all binding curves with an R^2^ value ≥ 0.95.

### 2.8. Cytotoxicity Assay

TZM-bl cells were seeded in 96-well plates (5 × 10^3^ cells/well). Serially diluted CLD or CLDmut protein was added to the cells. Following 72 h cultivation, MTT (Beyotime, Shanghai, China) was added to the culture medium to yield a final concentration of 0.5 mg/mL and incubated for 4 h at 37 °C in a CO_2_ incubator. The supernatants were carefully removed, and DMSO (200 μL) was added and mixed. After incubation for 10 min, absorbance at 570 nm (reference wavelength of 630 nm) was measured using a microplate absorbance reader (Tecan sunrise, San Jose, CA, USA). The cell viabilities were calculated using GraphPad Prism software (version 7.0, San Diego, CA, USA).

### 2.9. Statistical Analysis

All experiments were repeated three times unless otherwise indicated, and the data were analyzed with GraphPad Prism software (version 7.0, San Diego, CA, USA). A two-tailed unpaired Student t-test was used for comparisons between the two groups. One-way ANOVA and the Newman–Keuls multiple comparison test were adopted to compare the difference among three or more groups. *p* < 0.05 was considered statistically significant.

## 3. Results

### 3.1. Production and Purification of CLD and CLDmut Proteins in 293F Cells

**Figure 1:**
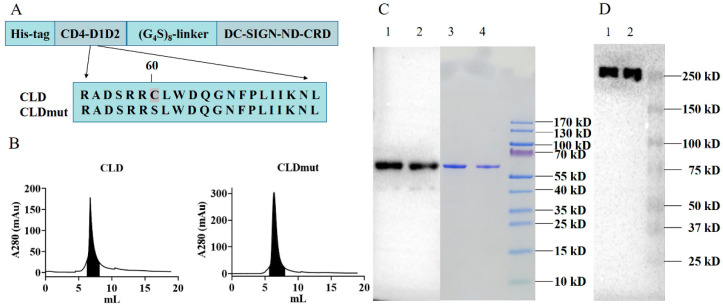
Production and purification of CLD and CLDmut proteins. (**A**) CLDmut was generated by introducing the C60S back mutation to CLD. (**B**) Biochemical characterization of the CLD and CLDmut. The CLD and CLDmut constructs were transfected, respectively, into 293F cells for protein expression in 300 mL FreeStyle^TM^ 293 Expression Medium. Proteins were purified with Ni-NTA followed by gel-filtration chromatography on Superdex^TM^ 75 Increase 10/300 GL. The graphs show elution profiles from the gel-filtration chromatography. The proteins in the shaded areas were collected. (**C**) SDS-PAGE, followed by Western blot analysis and Coomassie blue staining analysis of CLD and CLDmut proteins eluted from SEC (Size exclusion chromatography) column under reducing conditions. Anti-DC-SIGN MAb was used for detection. Lanes 1 and 3 represented elution samples at the peak of CLDmut (shaded area), and lanes 2 and 4 represented elution samples at the peak of CLD (shaded area). (**D**) Native-PAGE and Western blot analysis of CLD (lane 1) and CLDmut (lane 2). One out of three independent experiments is shown.

We previously demonstrated that fusion protein CLDs consisting of the D1D2 domain of CD4 and the neck domain (ND) and carbohydrate-recognition domain (CRD) of DC-SIGN produced in *E. coli* could efficiently inhibit HIV-1 infection in vitro and ex vivo. The construct had a Ser-to-Cys mutation (S60C) at amino acid 60 of CD4, which could form an interchain disulfide bond with gp120 to enhance binding affinity [18,24]. In this study, we further designed and eukaryotically expressed the CLD fusion proteins (all contain the S60C mutation) with a linker of 7, 8, or 9 Gly_4_Ser repeats, showing that the fusion protein with 8 Gly_4_Ser repeats was the most effective against two HIV-1 laboratory-adapted isolates BaL and NL4-3 (Supplementary Figure S1). The construct with 8 Gly_4_Ser repeats produced by mammalian cells was named CLD thereafter. As shown in Figure 1A, the CLD fusion protein consists of 6 × His-tag, the D1D2 domain of CD4, the linker of 8 Gly_4_Ser repeats, and the neck domain (ND) and carbohydrate-recognition domain (CRD) of DC-SIGN. While CLD carries the mutation S60C of CD4, CLDmut was generated by back mutating Cys to Ser at amino acid 60 of CD4. Both CLD and CLDmut proteins were subsequently expressed in mammalian 293F cells, purified by Ni-NTA, and followed by gel-filtration chromatography on Superdex^TM^ 75 Increase 10/300 GL (Figure 1B). The proteins in the shaded areas were collected. The BCA results showed that the obtained CLD and CLDmut were 256.7 µg and 881.6 µg, respectively. Reducing SDS-PAGE, followed by Coomassie staining and Western blot, showed that the purified CLD and CLDmut proteins were obtained, and the purity of the proteins was >95% as analyzed by Image J software (Figure 1C). Native-PAGE and Western blot showed that the purified CLD and CLDmut proteins appeared to be tetramers (Figure 1D), which were in agreement with the tetramerization characteristics of the DC-SIGN ND [18]. The predicted molecular mass of CLD and CLDmut tetramer was around 240 kDa. Additionally, an MTT assay was conducted to test the cytotoxicity of the proteins, and the results showed that CLDmut demonstrated no significant toxicity to cells up to 100 µg/mL (Supplementary Figure S6).

### 3.2. CLD and CLDmut Proteins Block the Infection of a Wide Range of HIV-1 Isolates

**Figure 2:**
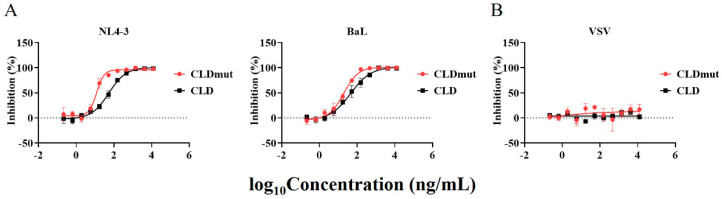
CLD and CLDmut proteins block the infection of HIV-1 but not VSV in TZM-bl cells. Purified CLD and CLDmut proteins were tested against the infection of HIV-1 NL4-3 and BaL (**A**) and VSV (**B**). Data shown are mean ± SD of three independent experiments.

To investigate the inhibitory activities of CLD and CLDmut, we assessed their neutralization efficiency against a range of HIV-1 strains in the TZM-bl cell line, while vesicular stomatitis virus (VSV) was used as a control to exclude nonspecific inhibition. We first tested two HIV-1 laboratory-adapted isolates, BaL and NL4-3, that use CCR5 and CXCR4 as co-receptors, respectively. Our data showed that both CLD and CLDmut exhibited high neutralizing activity against both HIV-1 isolates, with CLDmut being 2- to 5-fold more potent than CLD. In details, the IC_50_s of CLD against BaL and NL4-3 were 49.06 ng/mL (0.82 nM; 0.20 nM) and 55.52 ng/mL (0.93 nM; 0.23 nM), respectively, whereas those of CLDmut were 20.53 ng/mL (0.34 nM; 0.09 nM) and 12.22 ng/mL (0.20 nM; 0.05 nM), respectively. The indicated molar concentrations were calculated based on monomer (former) and tetramer (latter), respectively (Figure 2A, Supplementary Table S1). In contrast, neither CLD nor CLDmut showed neutralizing activity against VSV (Figure 2B). These results together indicate that CLD and CLDmut proteins potently inhibit HIV-1 infection in TZM-bl cells regardless of co-receptor usage, with CLDmut being more potent.

**Figure 3:**
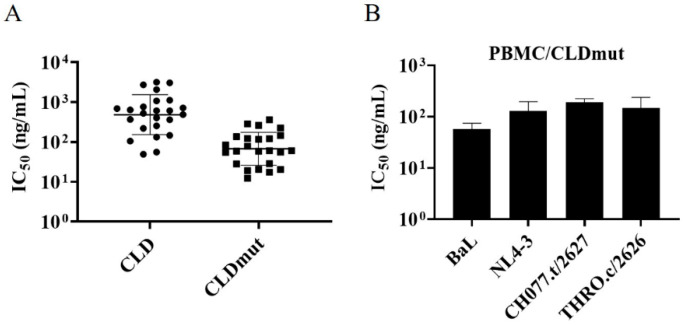
CLD and CLDmut proteins block the infection of a wide range of HIV-1 isolates in TZM-bl cells or PBMCs. (**A**) The IC_50_s of CLD and CLDmut proteins against 24 HIV-1 isolates in TZM-bl cells. (**B**) The IC_50_ of CLDmut proteins against 4 HIV-1 T/F isolates in PBMCs. Data shown are geometric mean ± SD of three independent experiments.

T/F HIV-1 viruses were isolated at an early stage after transmission. They are considered to be representative of the viruses that establish infection and, therefore, are more physiologically relevant than viruses isolated in chronic infection for use in prophylaxis studies [30,31]. Given that B and C subtypes are the main circulating isolates [32,33,34,35], we, therefore, chose 6 HIV-1 subtype B T/F isolates and 16 HIV-1 subtype C T/F isolates to conduct subsequent experiments. As shown in Figure 3, Supplementary Figures S2 and S3, and Supplementary Table S1, both CLD and CLDmut demonstrated potent neutralizing activities against the tested isolates. In particular, CLDmut had an IC_50_ of less than 100 ng/mL against 13 out of 22 isolates, whereas CLD had IC_50_ over 100 ng/mL against all of the 22 isolates. In general, CLDmut was at least 3-fold more potent than CLD, and the ratios of IC_50_ (CLD)/IC_50_ (CLDmut) were in the range of 2.39 to 34 (Supplementary Table S1), highlighting that CLDmut is more potent than CLD in neutralizing a wide range of HIV-1 isolates, in particular the T/F strains. The IC_50_s of CLDmut and CLD against the 24 HIV-1 isolates are shown in Figure 3A, indicating that CLDmut has a more potent and broader neutralizing activity.

Having demonstrated that CLDmut had stronger neutralization capability than CLD in TZM-bl-based assay, we next assessed whether the results are reproducible in primary cells. PBMCs derived from multiple donors were prepared, and the inhibitory activity of CLDmut against BaL, NL4-3, CH077.t/2627, and THRO.c/2626 was then examined. In accordance with the data from TZM-bl cells, CLDmut showed potent neutralizing activity in PBMCs against the tested HIV-1 isolates (Figure 3B, Supplementary Figure S4). The IC_50_s of CLDmut against these HIV-1 isolates were 56.34 ng/mL (BaL: 0.94 nM; 0.24 nM), 126.70 ng/mL (NL4-3: 2.11 nM; 0.53 nM), 190.40 ng/mL (CH077.t/2627: 3.17 nM; 0.79 nM), and 132.60 ng/mL (THRO.c/2626: 2.21 nM; 0.55 nM), respectively. The indicated molar concentrations were calculated based on monomer (former) and tetramer (latter), respectively (Supplementary Table S1).

### 3.3. CLDmut Has More Potent or Comparable Neutralizing Activity against HIV-1 in Comparison with bNAbs

**Figure 4:**
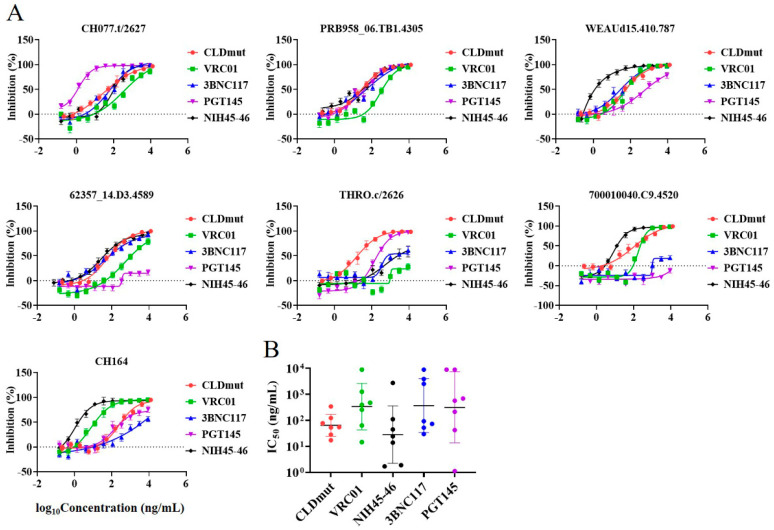
Comparison of the HIV-1 inhibitory activity of CLDmut protein with HIV-1 bNAbs. (**A**) CLDmut protein and bNAbs were tested against seven HIV-1 subtype B and C isolates in the TZM-bl neutralization assay. Data shown are mean ± SD of three independent experiments. (**B**) The above IC_50_s means were calculated and compared. Geometric means ± SD are indicated.

As demonstrated above, the results in both TZM-bl cells and PBMCs indicated that CLD and CLDmut could potently neutralize a wide range of HIV-1 subtype B and C isolates, including T/F strains, regardless of coreceptor usage. Given the broad and potent neutralizing activity of CLDmut against HIV-1, we next investigated whether CLDmut had comparable neutralizing activity as bNAbs. As shown in Supplementary Figure S5, we initially compared several HIV-1 neutralizing antibodies targeting different epitopes, including CD4bs-targeting NAbs (VRC01, NIH45-46 and 3BNC117), V1/V2-targeting NAbs (PG16 and PGT145), and V3-targeting NAbs (447-42D, 3869, PGT121 and 10-1074). A total of 4 potent bNAbs (VRC01, NIH45-46, 3BNC117, and PGT145) were eventually selected for subsequent experiments, and the maximum assay concentration was set as 9 μg/mL. The results showed that CLDmut had comparable or better neutralizing activity than the tested bNAbs against the panel of HIV-1 isolates (Figure 4A,B). In addition, CLDmut had broader neutralizing activity compared to some of the tested bNAbs. As shown in Figure 4A, THRO.c/2626 appeared to be relatively resistant against CD4-bs targeting bnAbs, whereas CLDmut could still potently neutralize THRO.c/2626. Compared to bNAb NIH45-46, which had exceptional breadth and potency as tested in this study, CLDmut demonstrated a similar broad neutralizing activity against the tested T/F isolates, with no significant difference (*p* = 0.4260) in IC_50_s geometric mean being observed between CLDmut (65.21 ng/mL) and NIH45-46 (28.72 ng/mL), as shown in Figure 4B and Supplementary Table S2. Taken together, these results suggest that CLDmut may have the potential to be used as an alternative to bNAbs or used in combination with bNAbs for the prevention of HIV-1 infection. 

### 3.4. CLDmut Has Strong Binding Affinity to HIV-1 gp140

**Figure 5:**
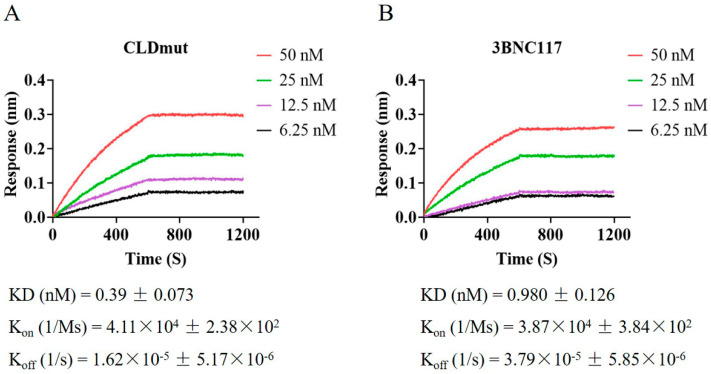
Binding of CLDmut protein to CN54 gp140. (**A**) CLDmut protein was conjugated with biotin at a molecular molar ratio of 1:3. Five µg/mL biotinylated CLDmut protein was coupled to SA biosensors and immersed in different concentrations of CN54 gp140 (50, 25, 12.5, or 6.25 nM) for association and dissociation. (**B**) 3BNC117 was coupled to AHC biosensors and immersed in different concentrations of CN54 gp140 (50, 25, 12.5, or 6.25 nM) for association and dissociation. The kinetics of binding was performed on a Forte-Bio Octet Red System. One out of three independent experiments is shown. KD: equilibrium (affinity) constant; K_on_: association rate constant; K_off_: dissociation rate constant.

We subsequently performed a direct binding analysis of CLDmut with HIV-1 Env. We used trimeric HIV-1 CN54 gp140, which contains gp120 and the membrane-external domain of the transmembrane subunit gp41 [36]. The bNAb 3BNC117 was used as a control as it targets the CD4-binding site on gp120 and has been shown to neutralize a number of HIV-1 strains [37]. The binding kinetics of CLDmut and 3BNC117 to HIV-1 CN54 gp140 were assessed on a Forte-Bio Octet RED system. A streptavidin biosensor immobilized with biotinylated CLDmut protein was immersed in different concentrations of CN54 gp140, with all protein molar concentrations being calculated based on monomers. We directly coupled 3BNC117 to AHC biosensors and immersed in different concentrations of CN54 gp140. As shown in Figure 5, the affinity of CLDmut (KD = 0.39 nM) was higher than that of 3BNC117 (KD = 0.98 nM). Compared with 3BNC117 (K_off_ = 3.79 × 10^−5^/s), CLDmut (K_off_ = 1.62 × 10^−5^/s) appeared to dissociate more slowly, indicating the formation of a more stable complex with gp140. CLDmut and 3BNC117 displayed comparable association rates, with a K_on_ being 4.11 × 10^4^/Ms and 3.87 × 10^4^/Ms, respectively. These results demonstrated that CLDmut had a stronger binding affinity to CN54 gp140 in comparison with the anti-HIV-1 bNAb 3BNC117. These results together may provide a molecular explanation for why CLDmut protein can potently block HIV-1 infection.

## 4. Discussion

In this study, we designed and expressed fusion protein CLDs with a linker of 7, 8, or 9 Gly_4_Ser repeats in mammalian cells, showing that the fusion protein with 8 Gly_4_Ser repeats (named CLD) was the most effective against 2 HIV-1 laboratory-adapted isolates BaL and NL4-3 (Supplementary Figure S1). A comparison of the fusion proteins bearing 7 Gly_4_Ser repeats produced in *E. coli* and mammalian cells, respectively, showed that the protein produced in mammalian cells was around 1.5-fold more potent in neutralizing HIV-1 BaL. Considering that the S60C mutation might not be essential for CLD expression in the eukaryotic system, we reversed the mutation and designated the construct CLDmut. We found that the designed fusion proteins CLD and CLDmut produced by mammalian cells potently inhibited the infection of a range of HIV-1 isolates, including T/F strains.

CLDs proteins expressed by 293F cells had stronger neutralizing activity against HIV-1 in comparison with those produced from *E. coli.* For instance, when tested against HIV-1 BaL, the IC_50_s of CLD (IC_50_ = 49.06 ng/mL) and CLDmut (IC_50_ = 20.53 ng/mL) from 293F cells were apparently lower than those (IC_50_ = 180~4326 ng/mL) of CLDs from *E. coli* [18]. In addition, the introduction of a Cys-to-Ser back mutation at the amino acid 60 of CD4 and the replacement of the linker of 7 Gly_4_Ser repeats with that of 8 Gly_4_Ser repeats likely also contributed to the enhanced antiviral potency of CLDmut. By exploring the broad-spectrum neutralizing activities of CLD and CLDmut, we found that both proteins potently inhibited the infection of two laboratory-adapted isolates and a range of subtype B and C T/F HIV-1 strains which represent the main circulating isolates with great physiological significance in infection [38]. These results were further confirmed in primary cells, revealing that CLDmut could potently block the infection of HIV-1 in PBMCs. CLDmut demonstrated no significant toxicity to cells up to 100 µg/mL, which was over 3 logs higher than its IC_50_s (Supplementary Figure S6). Further improvement of CLDmut may render it potentially useful in prophylaxis or therapeutics.

A number of bNAbs have been identified to decrease plasma HIV-1 RNA levels in HIV-1 infected individuals, such as the CD4-binding site-specific antibodies VRC01 and 3BNC117 [12,39], the V2-apex-specific antibodies PGDM1400, and the V3-glycan-specific antibodies PGT121 and 10-1074 [40,41,42,43]. In our study, CLDmut had comparable or better neutralizing activity than the tested bNAbs. CLDmut also demonstrated broader neutralizing activity, whereas some of the bNAbs could only neutralize a limited number of T/F HIV-1 isolates. It is known that HIV-1 resistance to bNAbs occurred in nearly all participants who received monotherapy [44]. A randomized, placebo-controlled, phase I clinical trial of PGT121 was reported recently, revealing that PGT121 was safe, well-tolerated, and had a brisk antiviral effect but induced virus resistance [43]. Although two randomized trials of bNAbs (VRC01) revealed that VRC01 did not prevent overall HIV-1 acquisition more effectively than placebo, strengthening the notion that a single bNAb may not be sufficient, analyses of HIV-1 isolates sensitive to VRC01 provided a proof-of-concept that bnAbs have the potential to prevent HIV-1 acquisition [45]. Moreover, isolates from VRC01 recipients were more resistant to VRC01 than those from placebo recipients. To improve the anti-HIV efficacy, efforts have been made, such as using a combination of bNAbs which target distinct epitopes to increase the breadth or work synergistically to promote a better performance. However, the use of bNAbs in combination would significantly increase the cost. Given the potent and broad neutralizing activity of CLDmut, CLDmut being used in combination with bNAbs may represent a promising strategy for the prevention and treatment of HIV-1.

The binding of gp120 to CD4 is virtually universal among HIV-1 isolates reported to date. Although a few CD4-independent HIV-1 strains were obtained by virus passage on CD4-negative, coreceptor-positive cells in tissue culture [46,47,48,49], such variants were rarely found in infected people [50,51] and were sensitive to neutralization by multiple antibodies that recognize different envelope glycoprotein epitopes [52]. Our fusion protein CLD/CLDmut contains CD4 and DC-SIGN components binding to different sites on gp120, and this may offer an advantage over other inhibitors which target CD4bs alone. Furthermore, the neck domain (ND) of DC-SIGN has been reported to lead to tetramerization through hydrophobicity, resulting in ND changing from β-rotation to α-superhelix, which can enhance protein affinity to corresponding ligands [17]. In our previous study [18], we constructed C35D composed of CD4 D1D2 and the CRD of DC-SIGN and C35ND composed of CD4 D1D2 and the ND and CRD of DC-SIGN. Compared with sCD4, the IC_50_ and IC_90_ of C35D decreased by 1.6-fold and 1.9-fold, respectively, indicating that the CRD effectively enhanced the antiviral activity. Compared with C35D, the IC_50_ and IC_90_ of C35ND both decreased by 3-fold. The enhancement might be due to ND, which could form a tetramer, resulting in a CLD containing four CRDs. In conclusion, the ND and CRD both likely played important roles in enhancing the antiviral activity of the fusion proteins.

In agreement, a heterotetrametric CD4-IgG2 inhibitor, consisting of CD4-V1V2 and the Fc portion of IgG2, was shown to inhibit HIV-1 envelope-mediated cell–cell fusion more effectively than monomeric sCD4 [53]. They also constructed a bispecific inhibitor, in which a VRC01 scFv was attached to PG16 IgG (VRC01_scFv_-PG16), and confirmed its in vitro neutralization potency against several HIV-1 strains [54]. Likewise, D1D2-Igαtp, consisting of CD4-D1D2, 18-amino acid secretory tailpiece (αtp) of IgA, and IgG1 heavy chain, was shown to have a much slower dissociation rate from gp120 than sCD4 [55]. Given that a single bNAb was not sufficient to prevent HIV-1 acquisition as reported in the VRC01 trial study, CLDmut, in combination with other HIV-1 bNAbs, may have a better potential in this regard.

Beyond a novel antiretroviral function, CLDmut may have additional potential. Firstly, recombinant adeno-associated viral vector (rAAV) has been reported to be a safe platform to offer long-term target gene expression [56,57,58]. Further studies are warranted to employ rAAV as a carrier to transduce the CLDmut gene in order to confirm its anti-HIV-1 potency in animal models. Secondly, the engagement of CD4 with gp120 induces the exposure of immunogenic epitopes on gp120. It is known that the CD4-gp120 complex immunization could promote the control of simian-human immunodeficiency virus (SHIV) infection [59,60]. In addition, HIV-1 Env in a CD4-bound conformation has been shown to be preferentially targeted by antibody-dependent cell-mediated cytotoxicity (ADCC)-mediated antibodies from HIV-1-infected individuals [61,62]. Given that DC-SIGN increases the affinity of HIV-1 Env interaction with CD4 [63], it may increase the exposure of CD4 binding sites and consequently promote the formation of more stable CD4-gp120 complexes. It will be interesting to determine in the future whether CLDmut-bound Env complexes can act as vaccine candidates to induce better neutralizing and/or ADCC-mediating antibodies.

## Data Availability

The primary data used to support the findings of this study are available from the corresponding author upon request.

## Supplementary Materials

The following supporting information can be downloaded at: https://www.mdpi.com/article/10.3390/v14071365/s1, Figure S1: CLD with a linker of 7, 8, or 9 Gly_4_Ser repeats blocks the infection of 2 HIV-1 laboratory-adapted isolates BaL and NL4-3 in TZM-bl cells. Figure S2: CLD and CLDmut block the infection of HIV-1 subtype B T/F isolates in TZM-bl cells. Figure S3: CLD and CLDmut block the infection of HIV-1 subtype C T/F isolates in TZM-bl cells. Figure S4: CLDmut protein blocks the infection of HIV-1 in PBMCs. Figure S5: Comparison of the HIV-1 inhibitory activity of CLDmut protein with several HIV-1 NAbs. Figure S6: MTT assay. Table S1: The IC_50_s of CLD and CLDmut proteins against HIV-1 infection. Table S2: The IC_50_s of CLDmut and HIV-1 bNAbs against HIV-1 infection. Original images: The original images of Western blot and Coomassie gel.
