# Supplementary material for: Fusion Proteins CLD and CLDmut Demonstrate Potent and Broad Neutralizing Activity against HIV-1
Source: Viruses. 2022 Jun 23;14(7):1365. doi: 10.3390/v14071365 (PMC9323411; doi:10.3390/v14071365)
Supplement: Supplementary file 1 [file viruses-14-01365-s001.zip › viruses-1651728-supplementary.pdf]

SUPPLEMENTAL MATERIAL

Fusion proteins CLD and CLDmut demonstrate potent and broad neutralizing activity against HIV-1

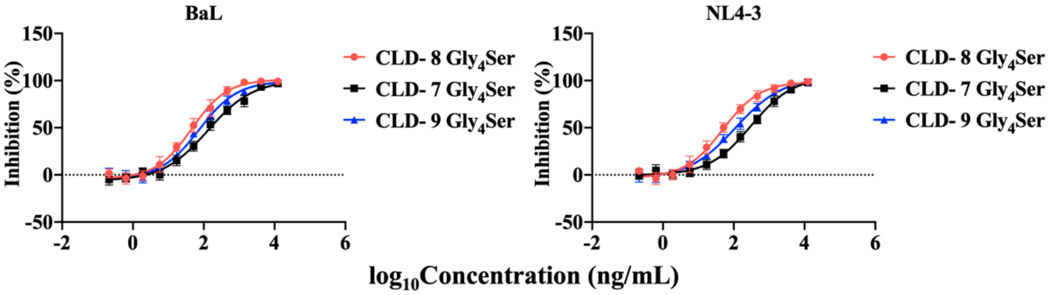

Figure S1. CLD with a linker of 7, 8 or 9 Gly4Ser repeats blocks the infection of two HIV-1 laboratory-adapted isolates BaL and NL4-3 in TZM-bl cells. Data shown are mean ± SD of three independent experiments.

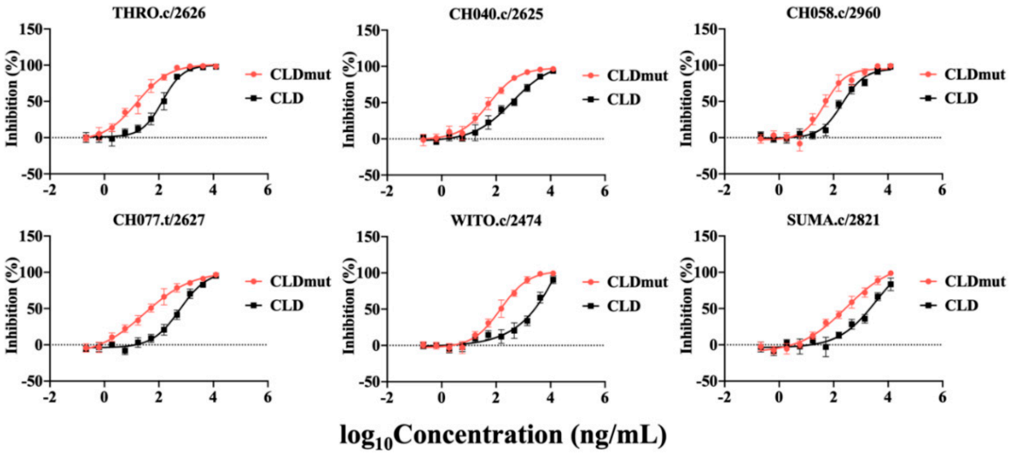

Figure S2. CLD and CLDmut block the infection of HIV-1 subtype B T/F isolates in TZM-bl cells. Purified CLD and CLDmut proteins were tested against 6 HIV-1 subtype B T/F isolates in TZM-bl cells. Data shown are mean ± SD of three independent experiments.

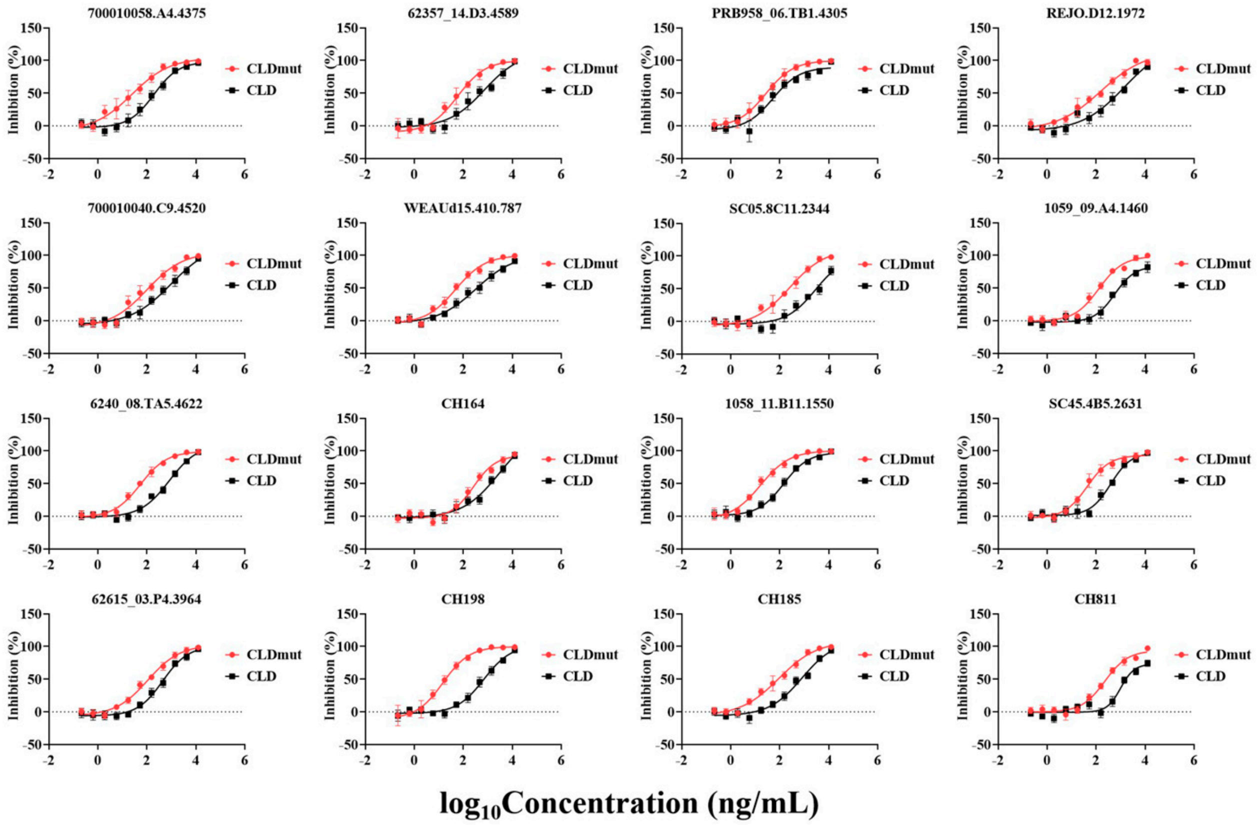

Figure S3. CLD and CLDmut block the infection of HIV-1 subtype C T/F isolates in TZM-bl cells. Purified CLD and CLDmut proteins were tested against 16 HIV-1 subtype C T/F isolates in TZM-bl cells. Data shown are mean ± SD of three independent experiments.

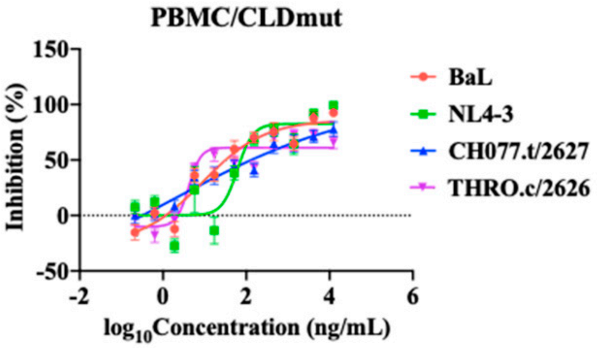

Figure S4. CLDmut protein blocks the infection of HIV-1 in PBMCs. CLDmut protein was tested against HIV-1 BaL, NL4-3, CH077.t/2627 and THRO.c/2626 in PBMCs.

Data shown are mean ± SD of three independent experiments.

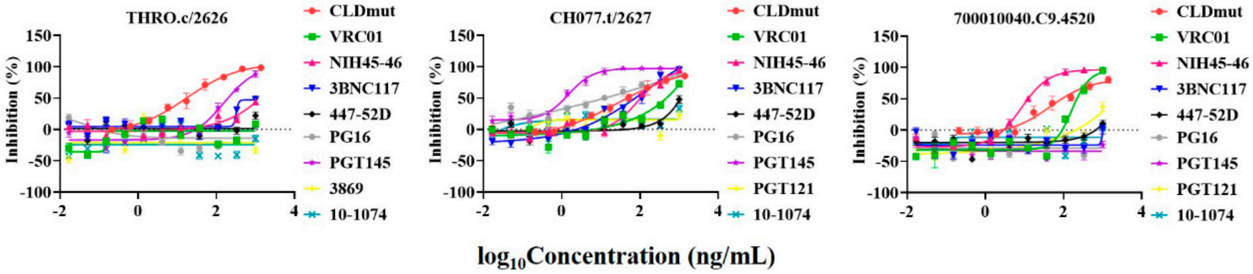

Figure S5. Comparison of the HIV-1 inhibitory activity of CLDmut protein with HIV-1 NAbs. CLDmut protein and NAbs were tested against 3 HIV-1 T/F isolates in the TZM-bl neutralization assay. Data shown are mean ± SD of three independent experiments.

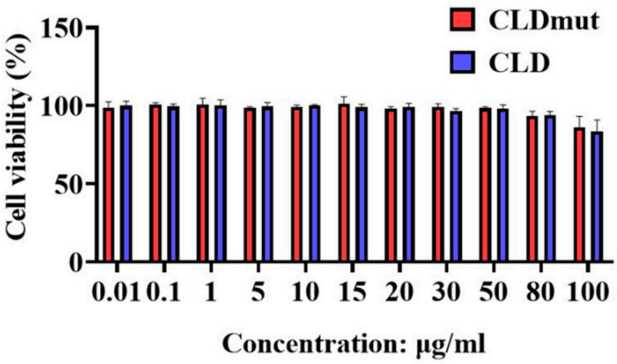

Figure S6. MTT assay. Cytotoxicity of different concentrations (from 0.01 to 100 μg/mL) of CLD and CLDmut proteins. The cytotoxicity of serially diluted CLD or CLDmut in TZM-bl cells was tested using MTT assay. Data shown were mean ± SD of three independent experiments.

Table S1. The IC_{50}s of CLD and CLDmut proteins against HIV-1 infection[a].

Cell	Strain	Subtype	IC_{50}[b] CLDmut (ng/mL)	95% CI[c]	IC_{50} CLD (ng/mL)	95% CI	Ratio[d]
TZM-bl	BaL	B	20.53	17.70-23.79	49.06	40.19-59.94	2.39
	NL4-3	B	12.22	10.35-14.51	55.52	48.15-64.00	4.54
	THRO.c/2626	B	17.42	14.28-21.24	132.00	111.00-156.70	7.58
	CH040.c/2625	B	60.39	50.08-72.85	361.40	293.00-446.10	5.98
	CH077.t/2627	B	54.83	42.74-70.48	637.30	530.90-765.7	11.62
	CH058.c/2960	B	60.32	46.13-79.66	253.60	206.30-312.80	4.20
	WITO.c/2474	B	144.80	120.10-174.50	2066.65	1592-2666	14.27
	SUMA.c/2821	B	262.33	203.70-338.20	2713.33	1534-2653	10.34
	62357_14.D3.4589	C	78.46	58.90-105.00	488.30	351.60-680.60	6.22
	REJO.D12.1972	C	117.45	89.20-154.70	759.99	558.30-1034	6.47
	700010040.C9.4520	C	120.60	87.24-167.40	607.80	477.00-775.70	5.04
	PRB958_6.TB1.4305	C	28.41	23.18-34.86	105.03	70.28-159.20	3.70
	WEAUd15.410.787	C	55.46	44.93-68.57	366.40	284.70-472.70	6.61
	SC05.8C11.2344	C	225.30	170.80-296.90	3169. 18	2320-4404	14. 07
	1059_09.A4.1460	C	136.50	112.60-165.80	1075.29	844.50-1377	7.88
	6240_08.TA5.4622	C	58.52	49.66-69.30	609.30	501.3-740.3	10.41
	700010058.A4.4375	C	28.16	21.05-37.63	219.10	173.00-278.00	7.78
	1058_11.B11.1550	C	19.11	15.83-23.12	146.53	121.00-177.50	7.67

	SC45.4B5.2631	C	59.29	47.02-75.14	403.27	328.30-495.80	6.80
	62615_03.P4.3964	C	121.60	99.61-148.50	521.70	420.90-647.60	4.29
	CH164	C	358.07	276.60-465.80	1121.61	881.80-1424	3.13
	CH185	C	83.25	66.17-104.70	713.69	559.20-912.10	8.57
	CH198	C	20.34	16.21-25.60	691.64	563.30-850.40	34.00
	CH811	C	283.42	229.20-351.30	3052.02	1757-3209	10.77
	Geometric mean		67.11		481.59		7.18
PBMCs	BaL	B	56.34	30.74-106.80	ND[e]	ND	ND
	NL4-3	B	126.70	70.63-260.90	ND	ND	ND
	CH077.t/2627	B	190.40	115.50-321.00	ND	ND	ND
	THRO.c/2626	B	132.60	53.69-359.9	ND	ND	ND

a. Data shown are mean of three independent experiments.

b. IC_{50}, 50 % inhibitory concentration.

c. CI, confidence interval.

d. Ratio, IC_{50}(CLD)/IC_{50}(CLDmut).

e. ND, not determined.

Table S2. The IC_{50}s of CLDmut and HIV-1 bNAbs against HIV-1 infection.[a]

		IC_{50}[b] (ng/mL)				
Strain	Subtype	CLDmut	VRC01	NIH45-46	3BNC117	PGT145
THRO.c/2626	B	17.42	> 9000	2792	2560	216.45
CH077.t/2627	B	54.05	480.72	112.20	73.90	1.13
62357_14.D3.4589	C	78.46	1272	45.18	93.93	> 9000
700010040.C9.4520	C	124.90	259.10	14.09	> 9000	> 9000
PRB958_6.TB1.4305	C	28.41	396.10	24.59	52.61	42.71
WEAUd15.410.787	C	55.46	64.49	1.72	29.97	700.20
CH164	C	345.03	14.78	1.91	3948	577.60
Geometric mean		65.21	341.20	28.72	372.52	319.82

a. Data shown are mean of three independent experiments.

b. IC_{50}, 50 % inhibitory concentration.

Original images

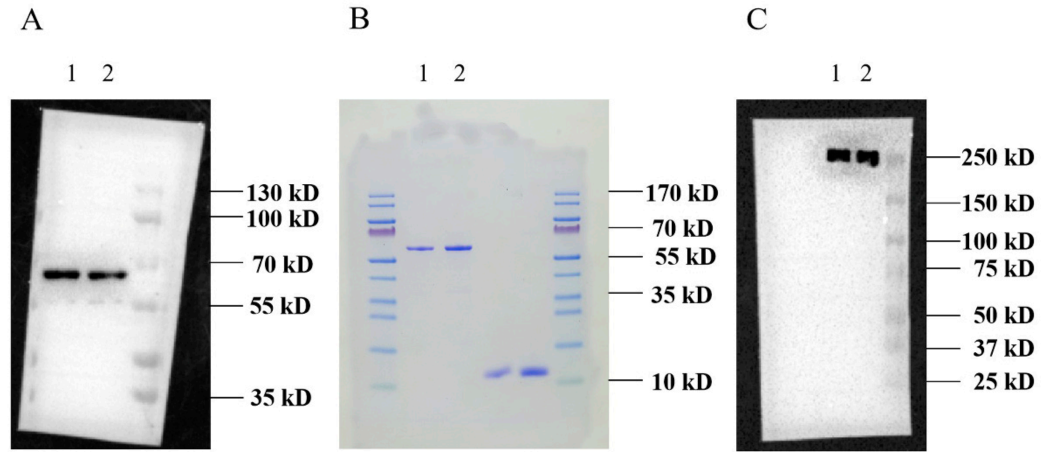

The original images of western blot and Coomassie gel. (A) SDS and western blot analysis of CLDmut (lane 1) and CLD proteins (lane 2) eluted from SEC column under reducing conditions. (B) SDS and Coomassie blue staining analysis of CLD (lane 1) and CLDmut (lane 2) proteins eluted from SEC column under reducing conditions. The two lanes on the right were unrelated proteins of another experiment. (C) Native-PAGE and western blot analysis of CLD (lane 1) and CLDmut (lane 2) proteins.
